# Grain Boundary Control of Organic Semiconductors via Solvent Vapor Annealing for High-Sensitivity NO_2_ Detection

**DOI:** 10.3390/s21010226

**Published:** 2021-01-01

**Authors:** Sihui Hou, Xinming Zhuang, Huidong Fan, Junsheng Yu

**Affiliations:** State Key Laboratory of Electronic Thin Films and Integrated Devices, School of Optoelectronic Science and Engineering, University of Electronic Science and Technology of China (UESTC), Chengdu 610054, China; shhou@std.uestc.edu.cn (S.H.); Zhuangxinming@std.uestc.edu.cn (X.Z.); 201511050103@std.uestc.edu.cn (H.F.)

**Keywords:** solvent vapor annealing, organic thin-film transistors, gas sensors, grain boundary, nitrogen dioxide

## Abstract

The microstructure of the organic semiconductor (OSC) active layer is one of the crucial topics to improve the sensing performance of gas sensors. Herein, we introduce a simple solvent vapor annealing (SVA) process to control 6,13-bis(triisopropylsilylethynyl)-pentacene (TIPS-pentacene) OSC films morphology and thus yields high-sensitivity nitrogen organic thin-film transistor (OTFT)-based nitrogen dioxide (NO_2_) sensors. Compared to pristine devices, the toluene SVA-treated devices exhibit an order of magnitude responsivity enhancement to 10 ppm NO_2_, further with a limit of detection of 148 ppb. Systematic studies on the microstructure of the TIPS-pentacene films reveal the large density grain boundaries formed by the SVA process, improving the capability for the adsorption of gas molecules, thus causing high-sensitivity to NO_2_. This simple SVA processing strategy provides an effective and reliable access for realizing high-sensitivity OTFT NO_2_ sensors.

## 1. Introduction

During the past decades, the global economy is developing rapidly, and people’s quality-of-life has also been greatly improved, leading to increased energy consumption. Most of the consumption comes from industrial, domestic, and transportation usage, which releases many poisonous and harmful gases into the air, resulting in environmental damage and health threats [1,2]. Nitrogen dioxide (NO_2_), as one of the toxic gases, mainly originates from the combustion of nonrenewable fossil fuels and is particularly dangerous. Long term exposure to NO_2_ will lead to serious respiratory diseases, including nose and throat irritation, emphysema, and bronchitis, even death at high concentrations (>100 ppm) by asphyxiation [3,4]. Besides, NO_2_ is one of the causes of acid rain and tends to accelerate the formation of microscopic particles, which seriously affects air quality [5]. Therefore, effective monitoring NO_2_ is of great significance to the production and life of humans. Compared to traditional technologies used for gases detection, such as optical, resistive, electrochemical, and chemiluminescent sensing, sensors based on organic thin-film transistors (OTFTs) have been widely concerned due to the advantages of room-temperature operation, low cost, simple fabrication, and high performance [6,7,8,9]. Besides, high signal-to-noise ratio can be obtained by the gate input of the OTFTs, which can amplify the sensing signal [10]. These properties make OTFT-based sensors attractive candidates for becoming the next generation gas sensors.

In recent years, several efforts have been made to explore new schemes for enhancing gas sensing performance of OTFTs. A considerable strategy is to design and synthesize novel sensitive organic semiconductor (OSC) materials [11]. By introducing specific functional groups, the interaction between the OSCs and gas analytes can be effectively enhanced, thus improving the stability, selectivity, and sensitivity of the devices [12]. Nevertheless, long time design and tedious synthesis process limit the development of this scheme [13]. The microstructure regulation of the OSC active layer is another effective strategy for improving sensing performance of OTFTs, and the point is to make the analytes interact with charge carriers more conveniently. For example, Chi et al. reported an NO_2_ sensors based on ultrathin crystalline 6,13-bis(triisopropylsilylethynyl)pentacene (TIPS-pentacene) films (7.5 nm), exhibiting a high sensitivity with a calculated limit of detection (LOD) of 20 ppb [14]. Except for decreasing the thickness of the active layer, forming a microporous structure is also an effective approach to improve the sensing performance, since the analyte molecules can diffuse to the channel directly through nanopores rather than via continuous and thick OSC films. Marks et al. fabricated OSC/insulator blend porous films by a breath figure patterning method, and the obtained gas sensors represented great responsivity of 280% to 10 ppm NO_2_ with fast response and recovery rate [15]. Another effective strategy is to control the crystallinity, grain boundaries, and roughness of the OSC films. Our group proposed an ultra-sensitive NO_2_ sensor by solvent selection method to balance the crystallinity and the grain boundary density in TIPS-pentacene films [16]. However, these methods are normally utilized during film-deposition, thus many of them are limited by the device structure, material type, and fabrication process.

Solvent vapor annealing (SVA) is a treatment process after film-deposition, which is often called post-annealing. During the SVA process, the films are exposed to a certain saturated solvent vapor atmosphere for a period. Compared to other post-annealing, including vacuum annealing, thermal annealing, and gas annealing, SVA process has been widely used in OTFTs because of its advantages of low processing temperature, slow re-organization process, simple set-up, and well-controllable [17,18,19,20]. Park et al. achieved high uniform and crystalline dip coated films by the chloroform SVA process with an average field-effect mobility of 11.6 × 10^−4^ cm^2^ V^−1^ s^−1^ [21]. Sun et al. utilized *o*-dichlorobenzene SVA to induce an anisotropic multiscale structure and obtained a high anisotropic thin film on an oriented polyethylene (PE) substrate with dichroic ratio of 7.1 [22]. Zhang et al. achieved highly oriented films by combining a magnetic field with SVA, which can control the polymer molecular arrangement, and the obtained device exhibited a 19-fold enhancement of electron mobility [23]. Overall, SVA is an effective way to control the molecular arrangement and morphology of OSC films. However, the applications of SVA process in OTFT-based gas sensors are still seldom studied.

Here, we implemented a simple SVA process for the microstructure regulation of OSC active layer to realize high-sensitivity OTFT-based NO_2_ sensors. The morphology of TIPS-pentacene film can be simply controlled by selecting different solvent vapors. The large density of grain boundaries was obtained by SVA process, thus enhancing the adsorption of the OSC films to NO_2_, which is essential for high-sensitivity gas sensors. The toluene SVA-treated devices exhibited an order magnitude enhancement of responsivity compared with the pristine devices. Furthermore, the sensors also possessed great recoverability, reusability, and selectivity with a LOD of 148 ppb.

## 2. Materials and Methods

### 2.1. Material Preparation

TIPS-pentacene (>99%, HPLC, glass transition temperature (*T*_g_) = 124 °C) and Poly(methyl methacrylate) (PMMA, *M*w = 120,000, *T*_g_ = 105 °C) were purchased from Sigma-Aldrich and used as received without further purification. The solvents, including 1,2-dichlorobenzene (1,2-DCB), anisole, toluene, o-xylene, and 1,3,5-trimethylbenzene (TMB), were purchased from Tokyo Chemical Industry Company. TIPS-pentacene was dissolved in 1,2-DCB with a concentration of 8 mg/mL, and PMMA was dissolved in anisole with a concentration of 10 wt.%. The solution was stirred on a magnetic stirring plate under room-temperature overnight to ensure complete dissolution.

### 2.2. Device Fabrication

**Figure 1:**
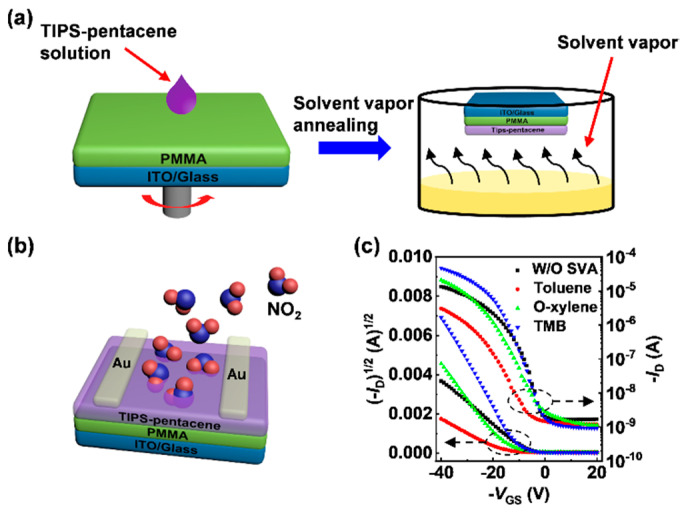
(**a**) Schematic illustrations of the SVA process for the TIPS-pentacene films. (**b**) The device architecture of the OTFT based NO_2_ sensor. (**c**) Transfer curves (*V*_DS_ = −40 V) for the pristine and SVA-treated OTFTs.

The schematic illustrations of the SVA process and the device structure of NO_2_ sensors are shown in Figure 1a,b, respectively. OTFTs were fabricated with a top-contact and bottom-gate structure. First, indium tin oxide (ITO) glass substrates were ultrasonically cleaned sequentially with acetone, deionized water, and isopropyl alcohol for 15 min each and dried at 80 °C in an oven for 1 h. PMMA, as a dielectric layer (400 nm), was spin-coated on these substrates at 1500 rpm for 60 s, and baked at 90 °C for 2 h. Subsequently, the TIPS-pentacene OSC films (40 nm) were spin-coated on the top of the PMMA at 3000 rpm for 60 s, and then annealed at 125 °C for 15 min to completely remove residual solvents. The SVA process was performed by exposing the spin-coated film to the solvent in a petri dish for 120 s (Figure 1a). The solvent vapor condition was obtained by releasing 2 mL of the solvent into the petri dish and waiting for 10 min to evaporate the solvent at room temperature (25 °C). After the SVA process, the films were rebaked at 125 °C for 10 min to remove the residual solvent, including the devices without the SVA process (the pristine devices) for the control variable. Finally, 40 nm gold source and drain electrodes were deposited on the OSC films by thermal evaporation with a shadow mask, and the channel length (*L*) and width (*W*) are 100 μm and 10 mm, respectively.

### 2.3. Measurement and Characterization

The OTFT based sensors were put inside an airtight chamber to evaluate the sensing performance, where dry air and gas analytes were introduced by a mass flow controller with a fixed flow rate of 100 sccm (standard cm^3^ min^−1^) to obtain a proper concentration of gases. The electrical characteristics of all the samples were tested by a Keithley-4200 semiconductor parameter analyzer under room temperature (25 °C), and the mobility (*μ*) was calculated in the saturation region via Equation (1) [24]:

(1) $$I_{\mathrm{DS}}\;=\;\left(\frac{WC_{i}}{2L}\right)\mu {\left(V_{GS}-V_{th}\right)}^{2}$$

where *I*_DS_ is the drain-source current., *V*_th_ and *V*_GS_ are threshold voltage and gate voltage, respectively, and *C*_i_ is the capacitance per unit area of the dielectric layer. The morphologies of the OSC films were characterized by Atomic force microscopy (AFM, MEP-3D-BIO, Asylum Research, Oxford, England) in a tapping mode. The crystallinity of the TIPS-pentacene films was measured by grazing incidence X-ray diffractometer (GIXRD, Bruker AXS GMBH D2 phaser, Bruker, Beijing, China).

## 3. Results and Discussion

The electrical characteristics of the OTFTs are shown in Figure 1c and Figure S1. It is clear that the electrical properties of the OTFTs are strongly affected by the SVA process. The corresponding electrical parameters were calculated and summarized in Table 1. Due to the re-organization of OSC films trigged by the SVA process [25], the on-current (*I*_on_, *V*_DS_ = *V*_GS_ = −40 V) increased from ~13.6 μA for the pristine device to ~47.7 μA for the TMB-treated device and ~20.8 μA for the o-xylene-treated device. In contrast, the toluene-treated device exhibited an opposite tendency falling to ~3.1 μA. Meanwhile, the *μ* of the devices showed a similar trend that the *μ* is ~0.036 cm^2^V^−1^s^−1^ for the pristine device, and increase to ~0.15 cm^2^V^−1^s^−1^ for the TMB-treated device and ~0.13 cm^2^V^−1^s^−1^ for the o-xylene-treated device, then decrease to ~0.015 cm^2^V^−1^s^−1^ for the toluene-treated device. Moreover, compared with the pristine devices, a negative shift of *V*_th_ and an enhancement in subthreshold slope (*SS*), which are normally related to the density of carrier trap at the interface between the OSC and the dielectric, occurs in the SVA-treated devices, indicating that the microstructure of the TIPS-pentacene films is charged by SVA processes, and related works also confirm this phenomenon [26,27].

**Figure 2:**
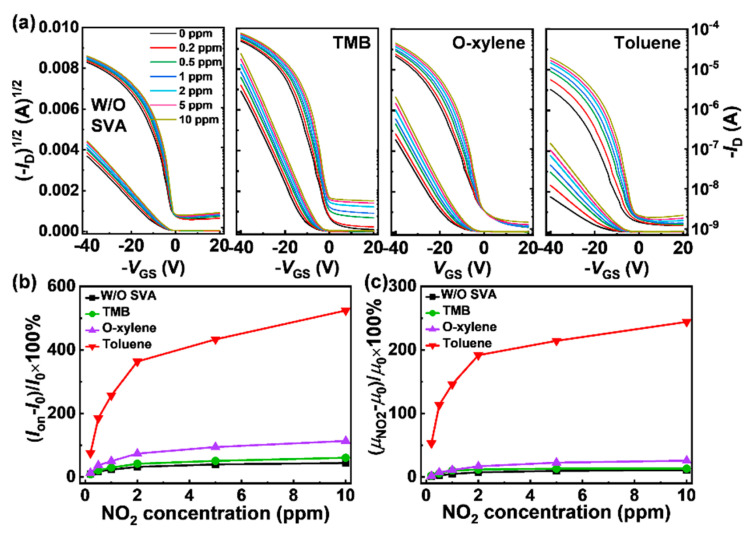
(**a**) Transfer curves of the OTFTs with different processes at room temperature (*V*_DS_ = −40 V). Variation of OTFT parameters of the sensors at different NO_2_ concentrations (0.2–10 ppm), (**b**) *I*_on_, (**c**) *μ* (*V*_GS_ = *V*_DS_ = −40 V).

Gaseous NO_2_ in the concentration of 0, 0.2, 0.5, 1, 2, 5, and 10 ppm were used as the analytes to characterize the sensing performance of the OTFTs. Typically, each cycle of *I*-*V* measurements was tested after introducing a certain concentration NO_2_ into the testing chamber for 2 min. The representative transfer curves of these TFTs under different NO_2_ concentrations are shown in Figure 2a. Compared to the pristine devices, the SVA-treated devices apparently exhibited improvement in sensitivity.

To further evaluate the sensing performance of these devices, the corresponding electrical parameters, including *I*_on_ and *μ*, for different NO_2_ concentrations were summarized and shown in Figure 2b,c. The responsivity of those parameters was used as the key factor and defined as (*P*_NO2_ − *P*_0_)/*P*_0_ × 100%, where *P*_NO2_ and *P*_0_ are the parameters of the OTFTs under NO_2_ and dry air atmospheres, respectively. As shown in Figure 2b,c, all the devices show increased responsivity of the parameters with increasing concentration of NO_2_ because of the electron acceptor effect of the oxidizing gas. When NO_2_ was introduced, a large amount of effective charge carriers was released, thus resulting in an increase of *I*_on_ and *μ* [16]. Note that the SVA-treated devices exhibited greater responsivity compared with the pristine devices under all testing concentrations, and the toluene-treated device showed the best response to NO_2_. Up to 10 ppm NO_2_, the responses of the pristine device were 44% for *I*_on_ and 11% for *μ*, whereas the SVA-treated devices exhibited significant variations in 60% (TBM), 114% (o-xylene), and 524% (toluene) for *I*_on_ and 13% (TBM), 26% (o-xylene), and 244% (toluene) for *μ*, respectively. Compared to the pristine device, the responsivity of the toluene-treated device exhibited a significant 11× enhancement for *I*_on_ and 21× enhancement for *μ*. Note, a sensitivity of 75% for *I*_on_ to 200 ppb NO_2_ is obtained by the toluene SVA process, while that of the pristine devices is only 9%, indicating the potential of the SVA process for improving sensing property with ultra-low NO_2_ concentration detection.

**Figure 3:**
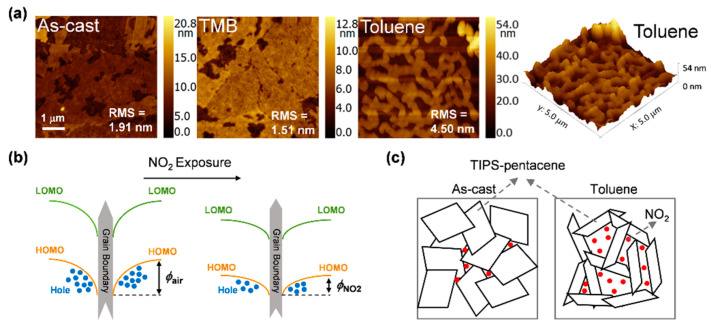
(**a**) AFM height images of the OTFTs. (**b**) Band diagram of the grain boundary before and after NO_2_ exposure. (**c**) Schematic of the two kinds of TIPS-pentacene films under NO_2_.

To explore how the SVA process enhances the sensing performance, AFM was employed to investigate the morphologies of the TIPS-pentacene films. Note, there was significant variation in the morphologies of the TIPS-pentacene films after SVA processes with a roughness of 1.51 nm for DMB and 4.50 nm for toluene, while that of the untreated film was 1.91 nm (Figure 3a). Besides, compared to the pristine device, the molecular morphology in the TMB-treated film becomes more uniform and bigger, while strip-shape grains are formed in the toluene-treated film, accompanied by large-area grain boundaries. The re-organization of OSC molecules is the key to the significant microstructural variations of films. During the SVA processes, the introduced solvent molecules will diffuse in and interact with the target OSC films, resulting in the plasticization and dilution effects, which could reduce the viscosity and the glass transition temperature, thus enhancing the mobility of OSC molecules and the fluidity of the films [20,28]. For TMB, the higher boiling point (168 °C) compared with post-annealing temperature (125 °C) provides enough time for the OSC domains to reorganize into low energy states. Therefore, the domain sizes are bigger than the untreated TIPS-pentacene film with smoother morphology. Instead, the low boiling point of toluene (110 °C) in the SVA process makes the OSC domain’s lack of adequate time to extend, thereby forming a strip microstructure with a large density of grain boundaries [29]. Moreover, the GIXRD measurements (Figure S2) indicated that the SVA processes could affect the crystallinity of the TIPS-pentacene films. In the TIPS-pentacene crystal structures, the peaks at 5° and 16° were correlated to the (001) and (003) reflections, respectively [16]. Herein, the increase of (001) reflections was observed for the TMB-processed TIPS-pentacene films, indicating that the crystallization and molecular orientation in the TMB-processed films were more uniform and ordered. This result is consistent with the OTFT electrical performance.

The transport of charge carriers in polycrystalline OSC films is mainly through migration in the crystals and hopping between adjacent grains [30]. The gaps existing in grain boundaries cause poor carrier transport and decrease of *I*_D_ and *μ*, which is consistent with previous experimental results presented in Table 1 [31]. As shown in Figure 3b, when oxidizing gas of NO_2_ was introduced, some trapped effective charge carriers in the grain boundaries will be released as a result of electron acceptation, leading to the diminution of the potential barrier (from *φ*_air_ to *φ*_NO2_) and thus increasing the number of hopping free holes. Therefore, an enlargement of the source-drain current and a higher *μ* can be obtained [16,32]. As shown in Figure 3c, compared to the untreated film, the toluene-treated film has a larger density of grain boundaries, in which a large number of NO_2_ can be absorbed and interaction with OSC molecules, thus exhibiting excellent sensing performance.

**Figure 4:**
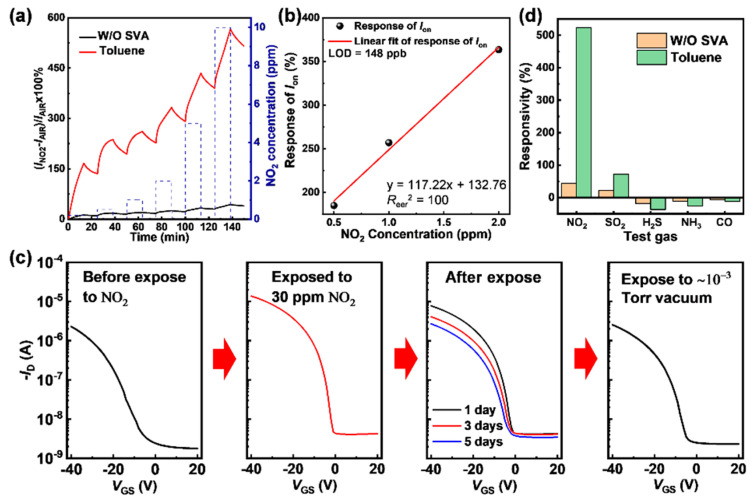
(**a**) The real-time responsivities of the devices to dynamic NO_2_ concentration at *V*_DS_ = *V*_GS_ = −40 V. (**b**) Calculation of limit of detection. (**c**) Transfer curves of the toluene-treated device when tested under different environments. (**d**) The response of the toluene-treated devices to various gases.

Real-time responsivities of the sensors were next measured under various NO_2_ concentrations (0.2, 0.5, 1, 2, 5 and 10 ppm) to further analyze the sensing performance. As shown in Figure 4a, the response of the toluene treated-device increases distinctly at all the NO_2_ concentrations. For instance, upon to 10 ppm NO_2_, the responsivity of the toluene-treated device showed 13× greater performance (566%) than the pristine device (44%). The response of the toluene-treated device to low concentration NO_2_ (200 ppb) was still as high as 167%, while that of the pristine device is 13%. Owing to the limitation of gas sources and the flow control system, 0.2 ppm was the lowest NO_2_ concentration that could be reliably used in these tests. Nevertheless, the LOD could be estimated by the root mean square deviation (RMSD) method [33]. As shown in Figure 4b, the responsivities of the toluene treated device to 0.5, 1, and 2 ppm NO_2_ were 185, 257, and 364%, respectively, which were extracted from Figure 2. The estimated LOD can be calculated by using Equation:

(2) $$Y_{\mathrm{LOD}}\;=\;\frac{3\times \sqrt{\frac{R_{eer}{}^{2}}{N}}}{S}$$

where *R*_eer_^2^ is the residual sum of squares, *N* is the number of data points, and *S* is the slope of the linear fit. The calculated LOD is 148 ppb, which is better than most reported sensors based on OTFT, demonstrating that such an SVA process is a significant scheme for high-sensitive gas sensors [6].

The device can realize multi-cycle detection for different NO_2_ concentrations, but a long recovery time was required (Figure S3), since the slow adsorption and desorption processes between OSCs and gas analytes [27,34]. Hence, the ability of recovery and multiple uses of the devices were evaluated, which is relevant to the device’s life and a critical factor for practical applications. As shown in Figure 4c, when exposed to 10 ppm NO_2_ for 10 min, the *I*_on_ of the device increased by five times, which was consistent with the previous response. After stored in air, the transfer curves gradually recover to their original state, and it is almost the same as before NO_2_ exposure on the 5th day. In addition, this recovery process can be accelerated by storing the devices in a vacuum. After placing the NO_2_ exposed device in the 10^−4^ Torr vacuum for a short time (1 h), the device quickly recovers to the state as stored in air for 5 days, indicating the physical adsorption of NO_2_ rather than an irreversible chemical interaction. Increasing the operating temperature is another practicable method, since a high temperature can accelerate the physical adsorption and desorption process [35]. Hence, these sensors could be reused for detecting a broad range of NO_2_ concentrations. In addition, the sensors also exhibited temporal stability to afford credible NO_2_ concentrations (Figure S4).

Finally, selectivity is another critical factor for practical applications. Herein, we tested both the pristine device and the toluene-treated device at a concentration of 10 ppm NO_2_, SO_2_, H_2_S, NH_3_, and CO atmosphere. Exposed to NO_2_, the *I_on_* responsivity increased from 44% to 524%, and that for other four gases are from 22% to 72% (SO_2_), −18% to −37% (H_2_S), −11 to −26% (NH_3_), and −6 to −12 (CO), respectively, indicating the higher density of grain boundaries enhance the interaction between OSCs and gas analyte molecules, not just NO_2_ (Figure 4d). However, the toluene-treated devices are at least 7× more sensitive to NO_2_ than the other four gases. In addition, the current responses for H_2_S, NH_3_, and CO is opposite compared to those of NO_2_ and SO_2_ as a result of different oxidizing/reducing properties [36]. Therefore, it can be deduced that these sensors can selectively identify NO_2_ from five kinds of common gas pollutants.

## 4. Conclusions

In summary, we developed a simple and effective SVA process to control the microstructure of the TIPS-pentacene films for high-sensitivity gas sensors. Compared to the pristine device, the toluene-treated device exhibited an order of magnitude enhancement of responsivity for NO_2_, besides with a LOD of 148 ppb. The large density of grain boundaries, which formed by re-organization of the OSC molecules during the SVA process, contributed to the improvement of sensing performance. The sensors also exhibited great recoverability, reusability, and selectivity. Thus, we believe this simple strategy opens up an innovative route for the fabrication of high-performance sensors.

## Figures and Tables

**Table 1:** Summary of the OTFT parameters.

Devices	*I*_on_ (10^−6^ A)	*µ* (cm^2^V^−1^s^−1^)	*V_th_* (V)	*SS* (V/dec)
W/O SVA	13.6 ± 2.2	0.036 ± 0.004	−7.1 ± 0.9	3 ± 0.6
TMB	47.7 ± 8.3	0.15 ± 0.03	−12.2 ± 1.2	3.8 ± 0.6
O-xylene	20.8 ± 4.7	0.13 ± 0.02	−13.5 ± 1.5	4.2 ± 0.7
Toluene	3.1 ± 1.2	0.015 ± 0.002	−14.8 ± 1.8	5.5 ± 1.1

Average of ≥5 devices. Measured under ambient conditions: relative humidity (RH) = 50–60%.

## Data Availability

No new data were created or analyzed in this study. Data sharing is not applicable to this article.

## Supplementary Materials

The following are available online at https://www.mdpi.com/1424-8220/21/1/226/s1, Figure S1: Output curves of the OTFTs with different SVA processes, Figure S2: GIXRD patterns of TIPS-pentacene films with different processes, Figure S3: The recovery curve of the toluene treated device to dynamic NO_2_ concentration at *V*_DS_ = *V*_GS_ = −40 V, Figure S4: Responsivity of the devices after stored in atmosphere for 3 weeks to different NO_2_ concentrations.
