# Supplementary material for: Grain Boundary Control of Organic Semiconductors via Solvent Vapor Annealing for High-Sensitivity NO2 Detection
Source: Sensors (Basel). 2021 Jan 1;21(1):226. doi: 10.3390/s21010226 (PMC7794992; doi:10.3390/s21010226)
Supplement: Supplementary file 1 [file sensors-21-00226-s001.pdf]

Support Information

Grain Boundary Control of Organic Semiconductors via Solvent Vapor Annealing for High-Sensitivity NO_2 Detection

Sihui Hou, Xinming Zhuang, Huidong Fan, Junsheng Yu*

State Key Laboratory of Electronic Thin Films and Integrated Devices, School of Optoelectronic Science and Engineering, University of Electronic Science and Technology of China (UESTC), Chengdu 610054, P. R. China

* Corresponding author. E-mail: jsyu@uestc.edu.cn.

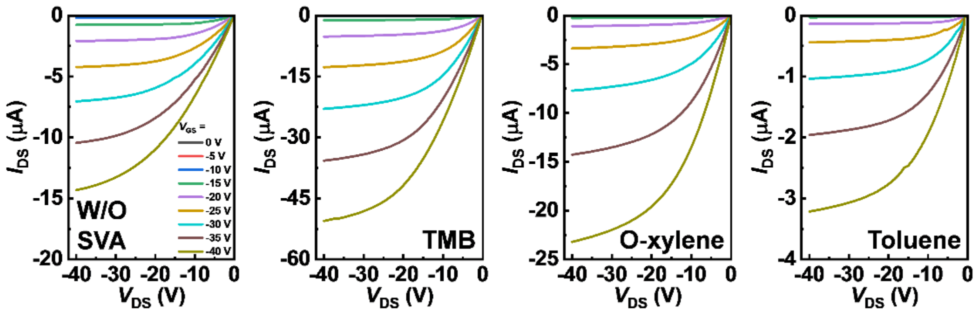

Figure S1. Output curves of the OTFTs with different SVA processes.

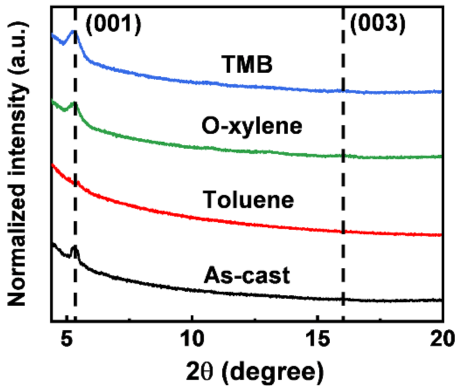

Figure S2. GIXRD patterns of TIPS-pentacene films with different processes.

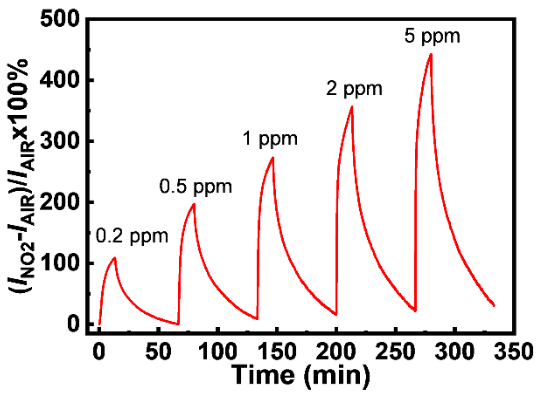

Figure S3. The recovery curve of the toluene treated device to dynamic NO_2 concentration at V_{DS} = V_{GS} = -40 V.

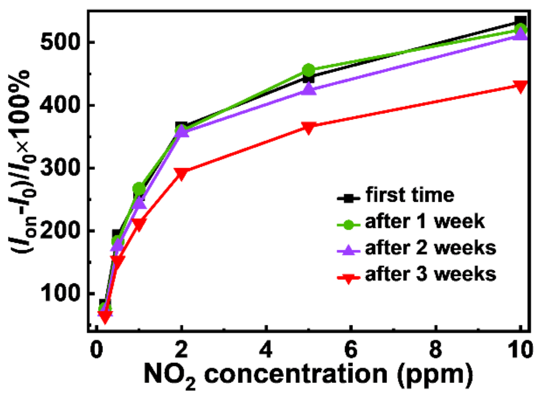

Figure S4. Responsivity of the devices after stored in atmosphere for 3 weeks to different NO2 concentrations.
